# One-Step Synthesis of Single-Wall Carbon Nanotube-ZnS Core-Shell Nanocables

**DOI:** 10.3390/ma9090718

**Published:** 2016-08-24

**Authors:** Yanli Zhang, Xiangming He, Li Wang, Jian Gao, Jianjun Li

**Affiliations:** 1 School of Materials Science and Engineering, Shenyang University of Chemical Technology, Shenyang 110142, China; ylzhang@alum.imr.ac.cn; 2 Institute of Nuclear & New Energy Technology, Tsinghua University, Beijing 100084, China; gaoj@tsinghua.edu.cn (J.G.); leejj@tsinghua.edu.cn (J.L.); 3 State Key Laboratory of Automotive Safety and Energy, Tsinghua University, Beijing 100084, China; 4 State Key Laboratory of New Ceramic and Fine Processing, Tsinghua University, Beijing 100084, China

**Keywords:** nanocables, SWCNT, ZnS, thermal reaction

## Abstract

Nanocables with a single-wall carbon nanotube (SWCNT) core and a ZnS shell were directly synthesized in one step through a thermal reaction method by using carbon, Zn, and FeS powder as starting materials. The as-fabricated nanocables were studied using scanning electron microscopy, transmission electron microscopy, and Raman spectroscopy. The nanocables have diameters of ~50 nm, lengths of several micrometers, and shell thickness of ~20 nm. TEM analysis revealed that the shell is polycrystalline wurtzite-type ZnS with good crystallinity, and the core of the nanocables is one to several SWCNTs. Raman results showed that the diameters of SWCNTs core are mainly distributed at 1.28 and 1.16 nm, with high quality and metallic character. A growth mechanism is proposed to explain the formation of the nanocables. This simple method may be applied to other SWCNTs-metal sulfide nanocables, which may have potential applications in photocatalysts, photocurrent, and other optical-electrical devices.

## 1. Introduction

Single-wall carbon nanotubes (SWCNTs) have unique electrical properties and can serve as a ballistic channel to carry current densities that are orders of magnitude higher than that of copper, which makes them one of the best candidates for application in nanoelectronics [1], transparent conductive films [2], conductive polymeric nanocomposites [3], and lithium ion batteries [4]. Except for pure SWCNTs, various SWCNT-based heterostructures are explored to widen the application of SWCNTs. Among them, SWCNTs’ surfaces being sheathed with another material and constructing a nanocable structure are quite attractive, because the one-dimensional nanocable configuration provides additional opportunities for the enhancement of the functionality of SWCNT 1D nanostructure, and the electronic interaction between SWCNTs and the external layer would be of great importance for the construction of various devices [5]. For example, individual SWCNT/Polypyrrole composite nanocables [6] and coaxial nanocables of SWCNTs surrounded by a Se shell have been synthesized, and they are envisaged to be useful in photonic devices [7].

Semiconducting nanostructures show attractive photoexcitation and photoresponse characteristics and have great potential applications in optical devices [8,9,10]. ZnS is an important II-IV semiconductor with a wide direct band gap of 3.6 eV [11]. ZnS nanostructures are widely explored in photo catalysis [12], photoluminescence [13], and sensors [14], etc. Reports reveal that the applications are hindered by low quantum efficiency and photocatalytic activity due to inefficient transfer and easy recombination of the generated electron/hole pairs. In order to improve this problem, multi-wall carbon nanotubes (MWCNTs) have been introduced as an electron channel to migrate the excited electron and alleviate the recombination of the e−/h+ pairs. Compared with MWCNTs, SWCNTs are less dense and lighter, so it is speculated that SWCNTs are more efficient. Above all, we propose a novel nanocable structure with SWCNTs as the core and ZnS as the shell (SWCNTs-ZnS nanocables) which has not yet been reported, and this structure may combine both the photo properties of ZnS and the electron properties of SWCNTs, thus display enhanced properties.

Usually, nanocables with CNTs as the core are synthesized by firstly dispersing CNTs into the solution to obtain dispersed tubes, and then wrapping them with inorganic shell materials. Nanocables with MWCNTs as cores were usually prepared with this method [15]. Due to the difficulty in dispersing SWCNTs (as they commonly exist in bundle form), reports on nanocables with SWCNTs as core are very limited. In addition, the dispersing step usually requires ultrasonic treatment and added surfactant [4], which would inevitably introduce defects and contaminations to SWCNTs, further affecting their intrinsic properties. Therefore, one-step synthesis of SWCNT nanocables with a perfect SWCNT core is urgently needed. In our previous work, coaxial nanocables of SWCNTs sheathed with amorphous silicon oxide were directly synthesized through a hydrogen arc discharge method [16], which indicated a feasibility of the direct synthesis of SWCNT-containing nanocables.

Herein, we develop a thermal chemical vapor deposition method to directly grow novel nanocables with SWCNTs as core and ZnS as shell. The growth and coating of SWCNTs are achieved in one step, which is not only simple but also avoids possible damage or contamination to SWCNTs that usually occurs in post-synthesis treatments. The morphology and microstructures of the nanocables are investigated by SEM, TEM, and Raman spectra. The growth mechanism of the nanocable is proposed.

## 2. Experimental

The synthesis of the SWCNT–ZnS nanocable was carried out by thermal reaction in a quartz tube reactor that was inserted into a horizontal tubular furnace. A mixture of graphite powder, iron powder (4.0 atm.%), zinc powder (1.0 atm.%), and FeS powder (1.0 atm.%) were used as the raw materials, which were well-mixed and cold pressed to a tablet with a diameter of 12 mm. The tablet was loaded into a ceramic boat and placed in the constant temperature region of the furnace center. High-purity argon gas was used as a carrier gas at a constant flux of 70 mL/min. The furnace was heated up from room temperature at a ramping rate of 10 °C min^−1^ to 1000 °C and maintained for 2 h. Then, the power source was pushed to the low temperature zone and the furnace was cooled down to room temperature. The product was collected on the downstream inner surface of the quartz tube. The morphology and structure of the as-prepared samples were characterized by a field emission scanning electron microscope (FEI Nova 400 Nano SEM, FEI, Hillsboro, TX, USA) and transmission electron microscope (FEI Tecnai G30, FEI, Hillsboro, TX, USA). Raman measurement was carried out on a Jobin Yvon LabRam HR800 Confocal Raman Spectrometer (HORIBA Jobin Yvon, Paris, France) with an excitation line of 632 nm.

## 3. Results and Discussion

**Figure 1:**
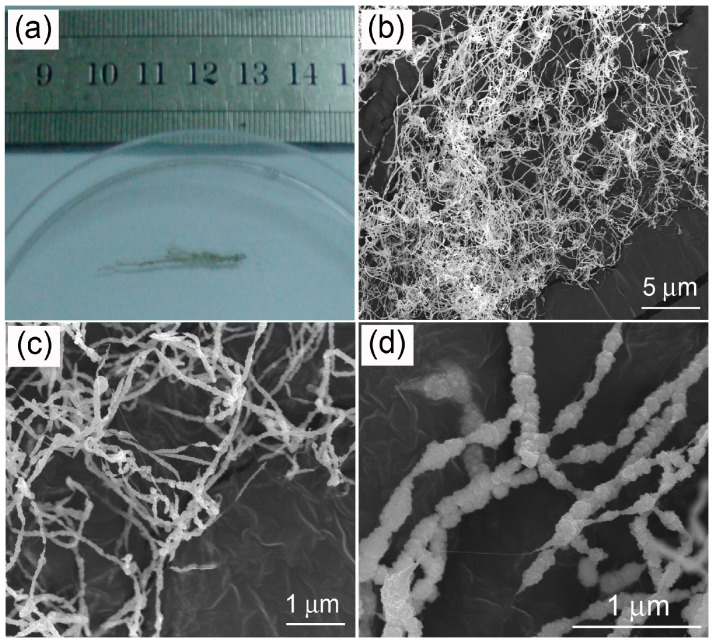
Optical image (**a**) and SEM images of the single-wall carbon nanotube (SWCNT)-ZnS nanocables: low magnification (**b**,**c**) and high magnification (**d**).

**Figure 2:**
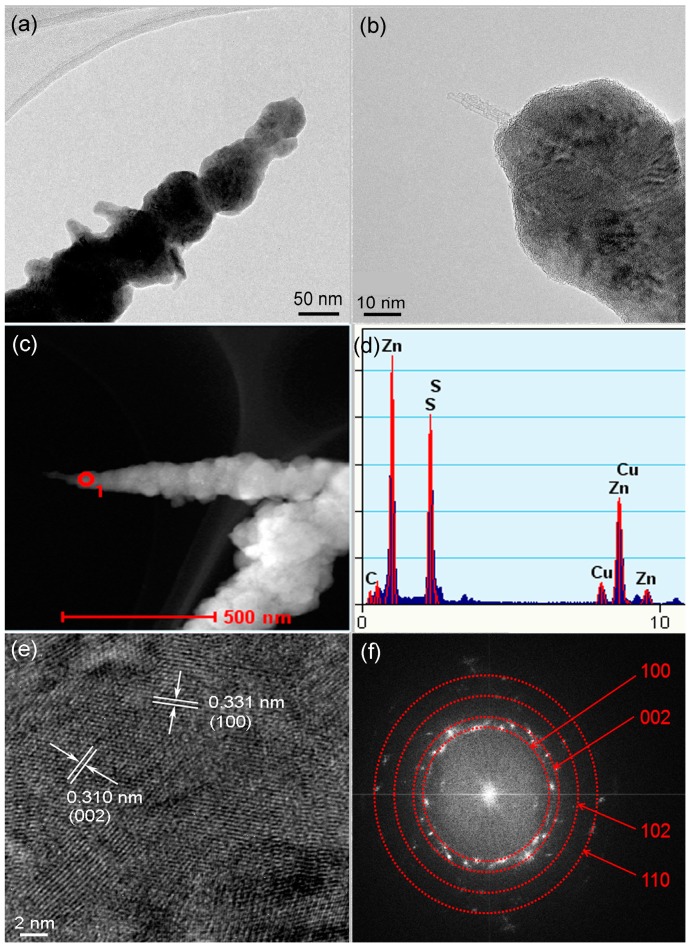
(**a**,**b**) TEM images; (**c**) high-angle annular-dark-field TEM (HAADF-TEM) image; (**d**) EDS spectrum; (**e**) high-resolution TEM (HRTEM image; and (**f**) Fourier transform pattern of the SWCNT–ZnS nanocables.

Figure 1a shows a typical optical image of the as-synthesized product, which is filamentous and about 3 cm long. Figure 1b–d show low and high magnification SEM images of the as-synthesized product. It can be seen that the sample is composed of pure entangled threadlike filaments with lengths varying from about one to tens of micrometers. TEM images in Figure 2a,b show the clearly core–shell structure of the product. The diameter of the filament is about 50 nm, and the thickness of the shell is in the range of 10–30 nm. The core is composed of one to several nanotubes with very tiny diameters of 1–2 nm, suggesting the formation of SWCNTs. A high-angle annular-dark-field TEM (HAADF-TEM) image of a nanocable is shown in Figure 2c, which also clearly shows core–shell contract. The EDS spectrum in Figure 2d confirms that the nanocable is mainly composed of Zn, S, and C, and the atom ratio of C:Zn:S is 18.2:40.8:41.0 atm.%. A high resolution TEM (HRTEM) image of the shell part is shown in Figure 2e. Two sets of lattice fringes can be seen, and the two inter-planar (d) spacings are 0.331 nm and 0.310 nm. Combining the EDS spectrum and the HRTEM image, we conclude that the core are SWCNTs and the shell is made up of polycrystalline wurtzite-type ZnS. These two lattice fringes correspond to the (100) and (002) lattice planes of ZnS. The corresponding Fourier transform pattern shown in Figure 2f contains several diffraction rings that can be indexed to (100), (002), (102), and (110) planes of ZnS. The obvious diffraction spots within these diffraction rings reveal a good crystallinity of the polycrystalline ZnS shell.

**Figure 3:**
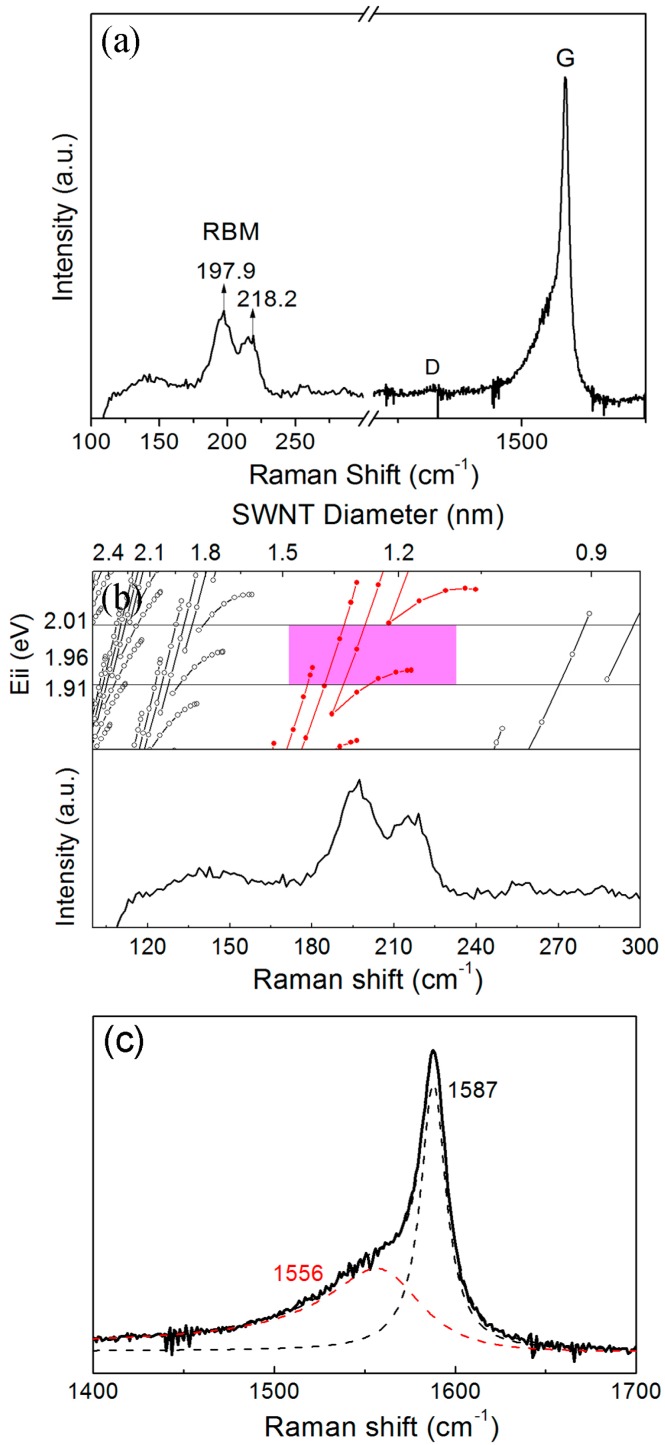
(**a**) Raman spectrum; (**b**) *E*_ii_-*d* plot or Kataura plot (upper panel) and radial breathing mode (RBM) region; and (**c**) G band of the SWCNT–ZnS nanocables.

Raman spectroscopy is used to further characterize the structure of the SWCNT–ZnS nanocables. A typical Raman spectrum is shown in Figure 3a. As we can see, the radial breathing mode (RBM) and G modes located at around 175–230 cm^−1^ and 1450–1600 cm^−1^ verify the presence of SWCNTs [17,18]. The RBM band contains two peaks centered at 197.9 and 218.2 cm^−1^. As the frequency of the RBM of SWCNTs is inversely proportional to the tube diameter, the diameter of SWCNTs corresponding to the above two peaks are calculated to be 1.28 nm and 1.16 nm, respectively, as calculated from the equation of ω (cm^−1^) = 254/*d* (nm) [19,20,21], where ω is Raman frequency, and *d* is the diameter of SWCNTs. The result is consistent with the TEM-observed diameters. In addition, as the Raman measurement originates from the resonance for a particular diameter of tube when the laser energy matches the electronic transition energy (*E*_ii_) of the tube [22,23], the electronic properties of the nanotube can be easily identified by Raman measurement. The upper of Figure 3b shows the diameter dependence of *E*_ii_—that is, a Kataura plot [24,25]. According to the resonant windows and the diameter, the two peaks located at 197.9 cm^−1^ and 218.2 cm^−1^ are attributed to metallic SWCNTs’ resonance. Therefore, plenty of metallic SWCNTs existed in the core of the nanocables, which will definitely facilitate the electron transport of the nanocables as photocatalysts. The presence of a large amount of metallic SWCNTs can also be verified by the Breit–Wigner–Fano (BWF) line shape [22,24,26] at 1556 cm^−1^ in the G band, shown in Figure 3c. The BWF line shape—originating from coupling of the discrete phonons to an electronic continuum—is a further sign of metallic SWCNTs. As we all know, the ratio of the intensity of the D/G band (*I*_D_/*I*_G_) is a measure of the defects present in SWCNTs, as the D band is due to out-of-plane vibrations attributed to the presence of structural defects [27,28]. *I*_D_/*I*_G_ of the nanocables is calculated to be 0.04, and the small value indicates that the SWCNTs sheathed in the nanocables have a good crystallinity.

**Figure 4:**
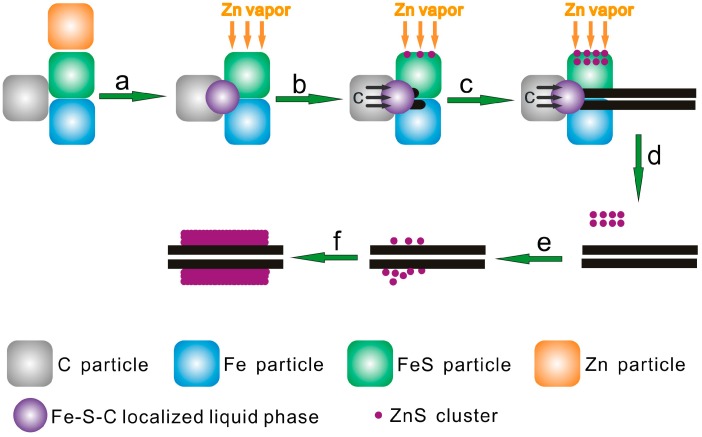
Schematic illustration of the formation process of the SWCNT–ZnS nanocables.

As described above, the SWCNT–ZnS core–shell nanocables are synthesized by a simple one-step thermal reaction. The possible growth process is proposed and schematically shown in Figure 4. It can be described as follows: (a) From the Fe–S–C phase diagram, the addition of carbon can result in a reduction of the melting temperature of pure FeS and Fe. Therefore, when the furnace temperature was increased to 1000 °C, Fe–S–C localized liquid zones (LLZs) are expected to be formed at certain points on the surface of FeS and Fe particles, where they are in contact with the adjacent carbon particles [29]. At the same time, Zn powder was converted into zinc vapor at its boiling point of 907 °C; (b) With the dissolving and diffusion of carbon particles into the Fe–S–C LLZs, carbon caps started to protrude out of the LLZs. Meanwhile, Zn vapor reacted with the adjacent FeS particles according to the reaction equation: Zn + FeS → Fe + ZnS, and thus ZnS nanoclusters were formed; (c) With the continuous diffusion of carbon atoms from carbon particles to the Fe–S–C LLZs, the carbon caps gradually grew into SWCNTs. The SWCNTs growth here does not involve any gas-phase reactant, and this suggests that the growth follows a solid–liquid–solid mechanism [30]. During the same period, more ZnS nanoclusters were formed; (d) Carried by the gas flow, SWCNTs left from the Fe–S–C LLZs and ZnS clusters left from the surface of FeS particles. Both of them were transferred downstream along with the gas flow; (e) During this stage, the ZnS clusters adsorbed onto the surface of SWCNTs, and nanocable structure was formed. The total surface energy can be reduced so this process is energetically favorable; (f) The growth of the nanocables ceased when they were transported to low temperature zone of the furnace by the gas flow. In the above synthesis, the chemical composition of the shell of the nanocables may be adjusted by replacing Zn with another metal element. By changing the weight ratio of Zn powder in the raw materials, the thickness of the shell may be tuned. The related studies are underway.

## 4. Conclusions

In summary, a novel SWCNT–ZnS core–shell nanocable was synthesized through a simple one step thermal reaction method. The nanocables are composed of a one-to-several SWCNTs core and wurtzite-type polycrystalline ZnS sheath. The nanocables are tens of micrometers in length and ~50 nm in diameter, with a shell thickness of ~20 nm. SWCNTs sheathed in the nanocables have a good degree of crystallinity and metallic character, which is expected to be useful for inhibition of the excition recombination in the ZnS sheath of the nanocables when being used as photocatalysts. The nanocables are also expected to be good candidates for photocurrent devices, due to the ability of SWCNTs to enhance the electron transfer of the semiconducting ZnS sheath. The preparation methodology suggests a general strategy to make other interesting SWCNT–metal sulfide core–shell nanocables.
